# Workers’ Exposure to Nano-Objects with Different Dimensionalities in R&D Laboratories: Measurement Strategy and Field Studies

**DOI:** 10.3390/ijms19020349

**Published:** 2018-01-24

**Authors:** Fabio Boccuni, Riccardo Ferrante, Francesca Tombolini, Daniela Lega, Alessandra Antonini, Antonello Alvino, Pasqualantonio Pingue, Fabio Beltram, Lucia Sorba, Vincenzo Piazza, Mauro Gemmi, Andrea Porcari, Sergio Iavicoli

**Affiliations:** 1 Italian Workers’ Compensation Authority—Department of Occupational and Environmental Medicine, Epidemiology and Hygiene, Via Fontana Candida 1, I-00078 Rome, Italy; ri.ferrante@inail.it (R.F.); f.tombolini@inail.it (F.T.); s.iavicoli@inail.it (S.I.); 2 Italian Workers’ Compensation Authority—Department of Technologies, via del Torraccio di Torrenova 7, I-00133 Rome, Italy; d.lega@inail.it (D.L.); al.antonini@inail.it (A.A.); a.alvino@inail.it (A.A.); 3 Laboratorio NEST—Scuola Normale Superiore and Istituto Nanoscienze—CNR, Piazza San Silvestro 12, I-56127 Pisa, Italy; pasqualantonio.pingue@sns.it (P.P.); f.beltram@sns.it (F.B.); lucia.sorba@nano.cnr.it (L.S.); 4 Laboratorio NEST—Scuola Normale Superiore and Italian Institute of Technology (IIT)—Center for Nanotechnology Innovation, Piazza San Silvestro 12, I-56127 Pisa, Italy; Vincenzo.Piazza@iit.it (V.P.); mauro.gemmi@iit.it (M.G.); 5 Italian Association for Industrial Research (AIRI), Viale Gorizia 25/c, I-00198 Rome, Italy; porcari@nanotec.it

**Keywords:** nanotechnologies, occupational safety and health, industrial hygiene, risk analysis, exposure measurement, harmonized tiered approach

## Abstract

With the increasing interest in the potential benefits of nanotechnologies, concern is still growing that they may present emerging risks for workers. Various strategies have been developed to assess the exposure to nano-objects and their agglomerates and aggregates (NOAA) in the workplace, integrating different aerosol measurement instruments and taking into account multiple parameters that may influence NOAA toxicity. The present study proposes a multi-metric approach for measuring and sampling NOAA in the workplace, applied to three case studies in laboratories each dedicated to materials with different shapes and dimensionalities: graphene, nanowires, and nanoparticles. The study is part of a larger project with the aim of improving risk management tools in nanomaterials research laboratories. The harmonized methodology proposed by the Organization for Economic Cooperation and Development (OECD) has been applied, including information gathering about materials and processes, measurements with easy-to-use and hand-held real-time devices, air sampling with personal samplers, and off-line analysis using scanning electron microscopy. Significant values beyond which an emission can be attributed to the NOAA production process were identified by comparison of the particle number concentration (PNC) time series and the corresponding background levels in the three laboratories. We explored the relations between background PNC and microclimatic parameters. Morphological and elemental analysis of sampled filters was done to identify possible emission sources of NOAA during the production processes: rare particles, spherical, with average diameter similar to the produced NOAA were identified in the nanoparticles laboratory, so further investigation is recommended to confirm the potential for worker exposure. In conclusion, the information obtained should provide a valuable basis for improving risk management strategies in the laboratory at work.

## 1. Introduction

In the last decade nanotechnologies (NTs) and nanomaterials (NMs) have seen rapid development worldwide, as seen in the growing numbers of R&D investments [1], nanotech enterprises (about 4000) [2], and nano-related products on the market (up to 1800) [3]. In the European framework NTs have been included among the six Key Enabling Technologies supporting industrial research and innovation in a wide range of sectors relevant for the European economy and as a crucial issue in the Horizon 2020 research program [4].

NMs are defined by the International Organization for Standardization (ISO) [5] as materials with any external dimension in the nanoscale (size range from approximately 1 to 100 nanometers); ISO specified that nano-objects and their agglomerates and aggregates (NOAA) are materials with one, two, or three external dimensions in the nanoscale including aggregates (strongly bonded or fused particles) and/or agglomerates (collection of weakly bound particles).

After 10 years of studies and research in this field, Maynard and Aitken [6] affirm that, alongside the emerging capabilities and increasing interest in the potential benefits of NT, these technologies may present novel and emerging risks for human health. Workers are among the first people exposed to the potential hazards since they are involved in all stages of the engineered NM life cycle, starting from research and development in the laboratory [7].

The toxicological literature suggests that multiple parameters may influence NMs health effects: their size, shape, particle number and mass concentration, surface area, aggregation and agglomeration, water solubility, and surface chemistry are the most important metrics in exposure studies [8,9,10].

Different strategies have been developed to assess airborne NMs and NOAA in the workplace, based on the integration of different aerosol measurement instruments providing NOAA exposure information in occupational settings [11,12,13]. Major drawbacks are related to the use of not-harmonized data collection methods and strategies and the lack of exposure-relevant records. Research efforts have focused on improving the release characterization towards a comprehensive exposure assessment [14].

The distinction of background from NOAA emission specifically related to the process plays a key role in any analysis of workers’ exposure. Two main approaches for background characterization are frequently used in measurement studies [11]:

(1) Far-Field (FF) approach: background is measured in a place not influenced by the process, in the same facility, but far from the workplace where NMs are produced. Some authors also consider the FF background measurements as a “spatial approach”, in which the difference between the background and workplace concentrations can be attributed to the work with the NOAA investigated. FF background measurements should be collected simultaneously with the NOAA measurements [15].
(2) Near-Field (NF) approach: based on monitoring before work, at the same location as the nano-workstation. The NF background is also defined as a “time-series” approach, assuming that the concentration determined when there is no ongoing work is the background concentration and any increases during work can be attributed to the process.

Further studies include measurements of outdoor background to better characterize the exposure scenario [12].

Therefore, international research efforts are aimed at defining standard methods for risk characterization and exposure assessment [16,17]. On the basis of a literature review of major exposure measurement initiatives, a harmonized, multi-metric tiered approach to measure potential exposure to NOAA in the workplace has been proposed by the Organization for Economic Cooperation and Development (OECD) [18]. This document defined a precise decision-support scheme, divided into three tiers of investigation: tier (1) information gathering, about materials, process and exposure scenarios; tier (2) basic exposure assessment based on moderate-cost screening methods, including real-time measurements and sampling with easy-to-use and portable instruments; tier (3) expert exposure assessment, using multi-metric real-time measurements, both area and personal sampling for gravimetric, chemical and morphological off-line analysis, to collect as much data as possible with the final aim of reaching reliable conclusions regarding the presence/quantification of airborne NOAA in the occupational environment.

The OECD document [18] suggested also using information from Control Banding (CB) risk management tools, with a view to qualitative risk analysis, by combining the hazard bands with the exposure potential, to define the level of control measures in the workplace [19,20]. In 2014 the ISO published a technical guide for checking and managing the risk related to NOAA in the workplace, based on the CB approach. In this specification, ISO encouraged exposure measurements, when feasible, since these are recognized as the best information for selecting the appropriate occupational exposure band [21].

The main objective of the present study was to propose a multi-metric approach for NOAA measurement and sampling, based on the harmonized and tiered OECD methodology; it was applied in three NOAA case studies with different shapes and dimensionality: graphene (G), a typical two-dimensional (2D) material, nanowires (NW) having a one-dimensional (1D) structure, and nanoparticles (NP) considered as having zero dimensionality (0D). The case studies were conducted at the NEST Laboratories in Pisa, Italy; these research facilities are very active in responsible development of nanotechnologies and have also conducted further studies to investigate the potential exposure of their personnel involved with NOAA [22].

The present study is part of a larger research project called “Nano-Lab” (www.nano-lab.it), in which field measurements will be employed to integrate and improve CB risk management for workers in the research laboratories involved with NOAA. Although all three OECD tiers have been conducted in line with the final aim of the project, the present study focuses on the results of the first and second tiers in order to assess the effectiveness of their application to the case studies.

## 2. Results

### 2.1. Information Gathering (Tier 1)

**Figure 1:**
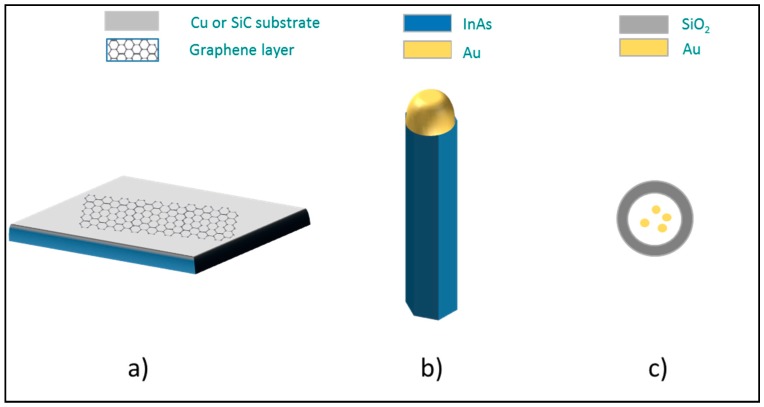
The three nano-objects and their agglomerates and aggregates (NOAA): two-dimensional (2D) graphene (G) (**a**), one-dimensional (1D) InAs nanowires (NW) (**b**) and zero-dimensional (0D) SiO_2_ core shell nanoparticles with Au nanostructure (NP) (**c**).

The first tier of our study focused on information gathering about materials, processes, and exposure scenarios. The case studies are related to NOAA with 2D, 1D, or 0D characteristics at the nanoscale (Figure 1): graphene (G), indium arsenide (InAs) nanowires (NW), and silica (SiO_2_) core shell nanoparticles containing an aurum (Au) nanostructure (NP). The NOAA physical–chemical characteristics such as shape, size, composition, production method and others, are reported in the technical data sheets (Table A1 in Appendix). The three NOAA were chosen for their characteristics (shape, dimensions, etc.) common in the nanosystems widely employed in research laboratories and including some peculiarities to distinguish the NOAA produced “on purpose” from the background.

**Figure 2:**
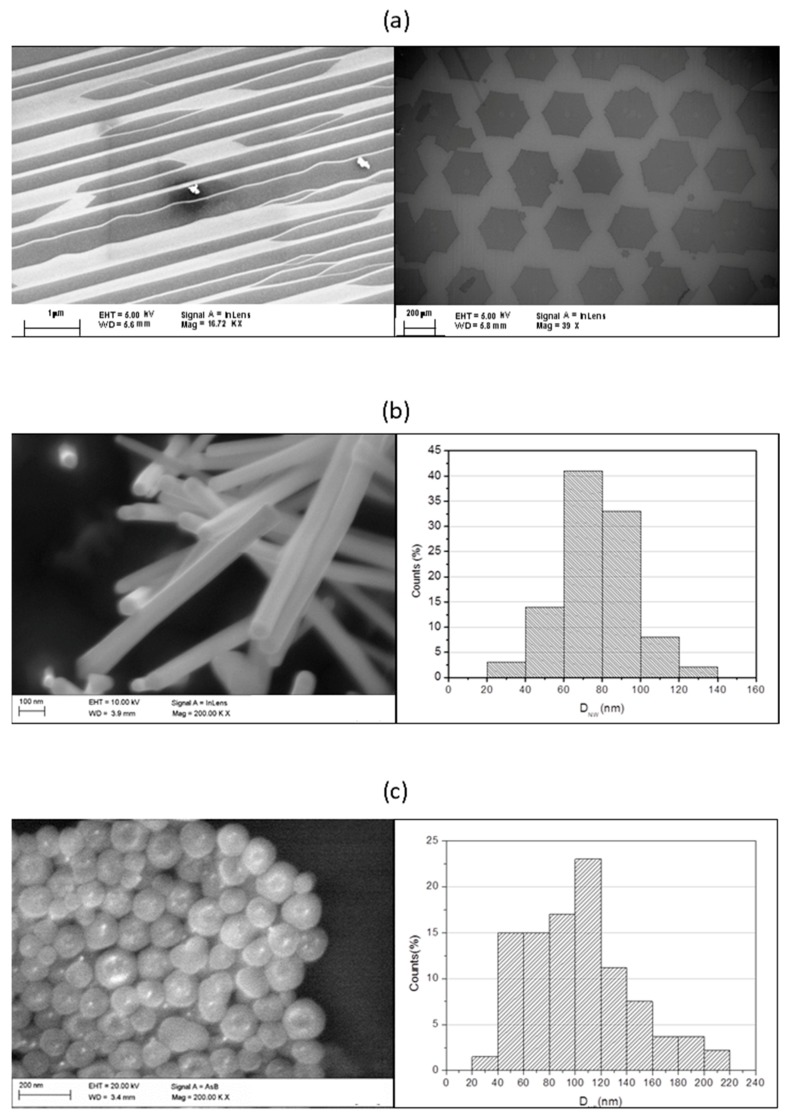
SEM images of: (**a**) graphene (G) deposited on SiC (**left** panel) and Cu (**right** panel) substrates, magnification 16.72 Kx and 39 x; (**b**) InAs nanowires (NW) and histogram of section diameter size distribution (D_NW_), magnification 200 Kx; (**c**) SiO_2_ core shell with Au nanostructures (NP) and histogram of diameter size distribution (D_NP_), magnification 200 Kx.

We used a high-resolution field emission gun scanning electron microscope (FE-SEM) equipped with an energy dispersive spectroscopy system (EDS) to collect morphological and chemical information on NOAA trial materials. Two SEM images of G are reported in Figure 2a: in the left-hand panel the darker areas are the G terraces grown on silicon carbide (SiC) substrate; in the right-hand panel the entire surface is covered by a monolayer of G grown on copper (Cu) foil. The typical lateral size of the graphene flakes lying on top of these substrates ranges from 10 μm to a few mm, according to the G data sheet in Table A1.

SEM images in Figure 2b show the InAs NW, with a typical hexagonal section and a gold nanoparticle on one of their extremities. The corresponding diameter size distribution, obtained measuring 100 NW with SEM Scandium software, was centered at about 80 nm, according to the NW data sheet (Table A1).

**Figure A1:**
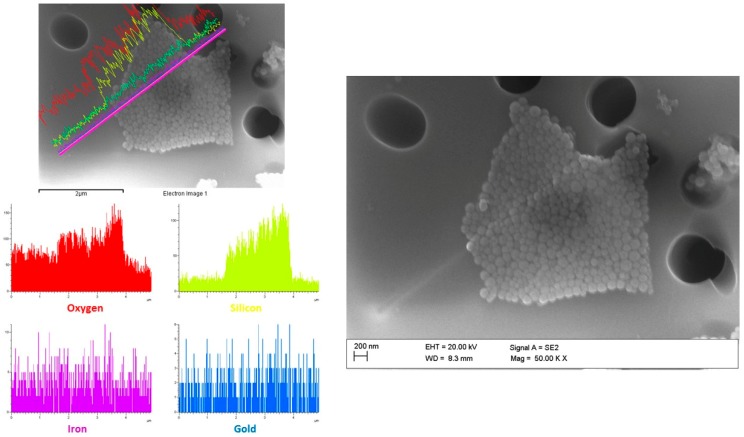
Nanoparticles (NP).

The SEM image in Figure 2c depicts the SiO_2_ core shells. The bright dots are generated by Au nanostructures embedded in the shell, even though the EDS analysis gave a low Au signal (see Figure A1). The diameter distribution, obtained measuring 150 NP, indicated that the most frequent diameter was less than 120 nm for SiO_2_ and a few nm for Au, according to the data sheet in Table A1.

From analysis of the information collected in this phase we concluded that in all three case studies the release of NOAA cannot be excluded, therefore further tier 2 analysis was needed, according to the OECD methodology [18].

### 2.2. Basic Exposure Assessment (Tier 2)

Tier 2 focused on the time series measurements of particle number concentration (PNC) with a hand-held condensation particle counter (CPC) and air sampling using a Sioutas personal impactor whose filters were analyzed off-line by SEM. Particular attention was directed toward any outlier in the time series measured in the workplace, in relation to the background levels. In these cases, to make the data more reliable, we compared PNC measurements carried out simultaneously by CPC and a fast mobility particle sizer (FMPS). Indoor climatic parameters were continuously monitored during all measurements and sampling. Further information about the measurement strategy and instrument features is reported in the Materials and Methods section.

**Figure 3:**
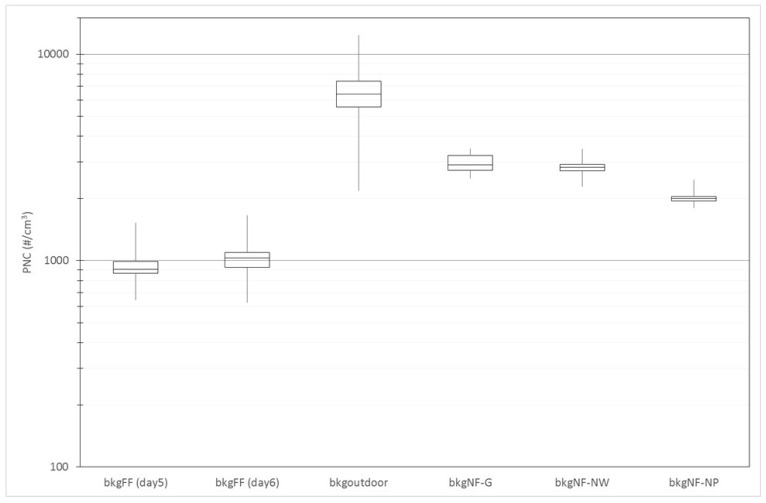
Box plots of far-field (FF), outdoor, and near-field (NF) background particle number concentration (PNC) measured by condensation particle counter (CPC) (#/cm^3^).

Figure 3 shows the median, minimum, maximum and percentiles of PNC measured by CPC related to FF (day 5–6 of the measurement campaign), outdoor (day 9) and NF background obtained in the G (day 3), NW (day 1) and NP (day 7) laboratories, before starting each process. The lowest edge of the box indicates the 25th percentile, the line inside the box marks the median, and the top of the box indicates the 75th percentile. Whiskers above and below indicate the lowest and highest values. All background measurements were taken with 1-s time resolution and the daily collection time was 7 h for FF and outdoor background and 15 min for each NF background in the G, NW and NP laboratories.

Box plots of FF background show an asymmetric distribution of PNC:

- on day 5 the median, at 907 #/cm^3^, was much closer to the lower edge whereas on day 6 the median, at 1029 #/cm^3^, was closer to the upper edge of the box;
- the distribution dispersions (interquartile distance on day 5 was 123 #/cm^3^ and on day 6 it was 164 #/cm^3^) were not dissimilar.

The outdoor background (bkg_outdoor_) box plot shows a symmetric distribution of PNC with the highest values (median 6420 #/cm^3^ and interquartile distance 1858 #/cm^3^). The NF background (bkg_NF_) box plots too show symmetric distribution; medians are 2829, 2904 and 1997 #/cm^3^ and the corresponding interquartile distances are 196, 493 and 106 for the NW, G and NP laboratories.

Mean PNC and standard deviation (σ bkg) of PNC measured by CPC in the FF, outdoor background air and in the NF of the G, NW and NP laboratories before the processes (bkg_NF-G_, bkg_NF-NW_ and bkg_NF-NP_ respectively) are summarized in Table 1. The type of background is described in the first column and the second column indicates the collection interval for each background measurement. The mean of 986 #/cm^3^, with the corresponding standard deviation of 167 #/cm^3^ related to two days of measurements, was considered as FF background (bkg_FFavg_).

All the NF average background levels at the beginning of each production process were higher than the FF background; bkg_FFavg_ may be the lowest because the room used for these measurements was a laboratory where no work was done during the data collection.

In the NW and G laboratories, although the mean (and median) bkg values of PNC are very similar, the corresponding variances are different; furthermore, in the G laboratory the NF σ bkg value is higher than the others. This last variability can be attributed to research staff passing through frequently for work-related activities in the laboratory and therefore near the CPC sampling point; this was observed in both the G and NW laboratories during the background data collection interval. In the NP laboratory the σ bkg_NF_ value was lower than in the NW and G laboratories.

Last, mean and σ bkg_outdoor_ are the highest recorded values, essentially influenced by environmental pollution, vehicles and motorcycles passing near the CPC collection point during the measurements.

**Figure 4:**
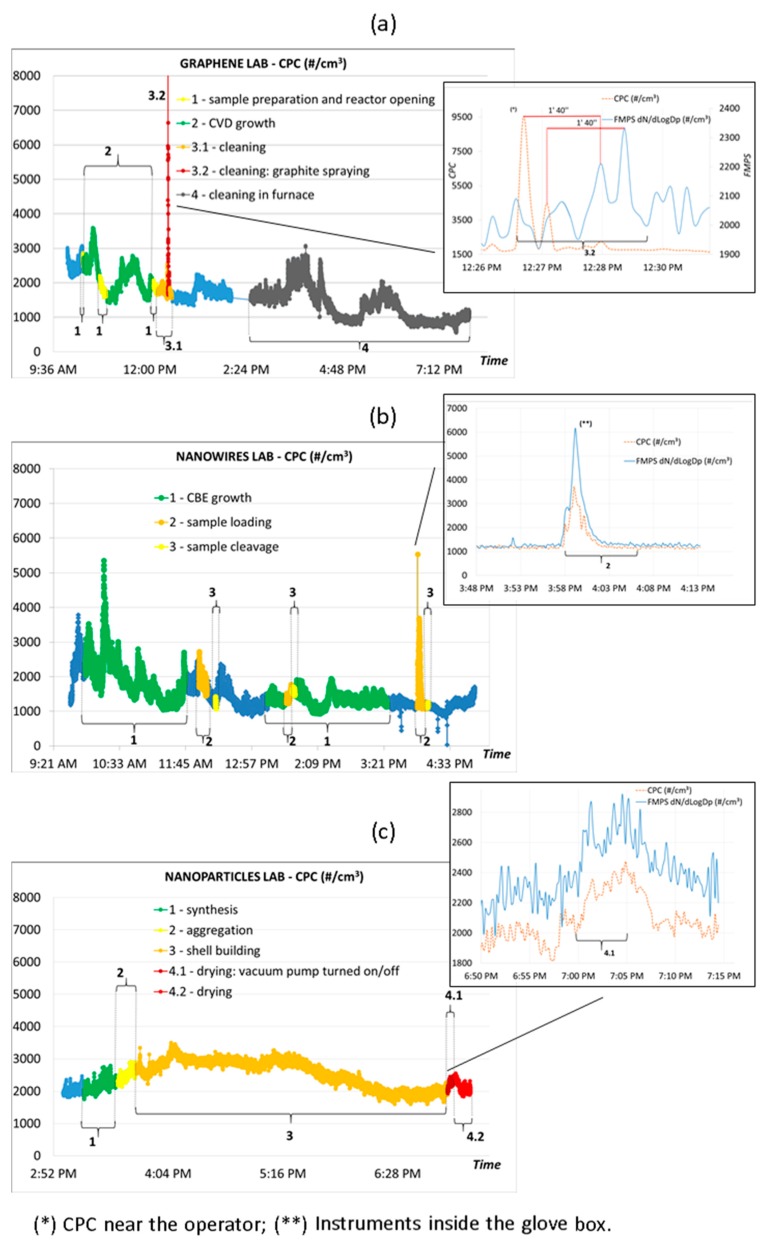
Time series of PNC (#/cm^3^) measured by CPC in the G (**a**), NW (**b**) and NP (**c**) laboratories. The insets show the corresponding fast mobility particle sizer (FMPS) measurements.

Time series of workplace PNC, measured on one day in each case study, summarizing all the process phases, are reported in Figure 4: day 4 for G, day 2 for NW and day 7 for NP laboratories. Details of each process phase with the related measurement and sampling activities are given in the Materials and Methods section. The different process phases are highlighted in different colors and with curly brackets. Total PNC obtained by FMPS (as in the insets of Figure 4) confirmed the values measured simultaneously by CPC in the three case studies.

In the G laboratory (Figure 4a), measurements started at 10:05 a.m. and ran up till the end of day 4. The inset shows the peak in the intensity signal measured simultaneously by FMPS and CPC instruments (10-s averages) during phase 3.2 of the graphite spraying on some reactor components during cleaning. According to the CPC measurement, the FMPS signal gives the same features with a delay of about 1 min and 40 s. This might be due to the different distances of the two instruments from the source of the graphite particles: FMPS was in the center of the room and CPC near the operator. The lower peak intensity measured by FMPS is probably due to the different dilution rates depending on the locations of the two instruments [23].

In the NW laboratory (Figure 4b), the measurements on day 2 started at 9:40 a.m. and finished at 5:09 p.m. The total PNC shown in the inset was simultaneously obtained by the CPC and FMPS instruments during the specific task of sample mounting/unmounting inside the glove box in phase 2. The lower signal intensity recorded by the CPC compared to the FMPS can be attributed to the different collection points of the two instruments inside the glove box (FMPS on the top and CPC on the floor). This experimental set-up meant that the CPC collection point was not fully hit by the streamlined airflow inside the glove box.

Figure 4c sets out the CPC time series measured in the NP laboratory, from 2:57 p.m. to 7:19 p.m. on day 7. In the inset the process phase 4.1 (drying vacuum pump turned on) is highlighted by comparison of the CPC and FMPS PNC measurements: in this case the lower intensity measured by FMPS may be substantially due to the instruments’ accuracy [24] and their different particle size ranges (CPC 10–1000 nm; FMPS 5.6–560 nm).

**Figure 5:**
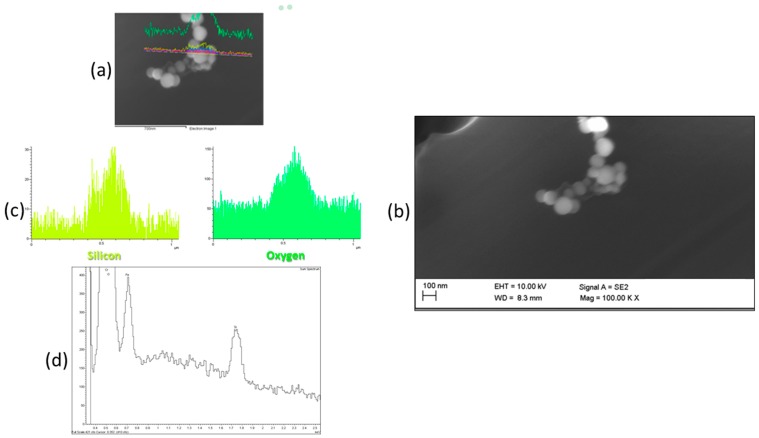
SEM image (**a**,**b**) and EDS spectrum (**c**,**d**) of SiO_2_ core shell NPs collected by the Sioutas (Stage D: 250–500 nm): Magnification 100 Kx, detector SE2, EHT 10.00 KV, WD 8.3 mm.

SEM images of the filters sampled by Sioutas worn by workers during each production process did not detect any materials attributable to those produced in the G and NW laboratories. The morphological investigations of D (250–500 nm) and backup (<250 nm) sampled filters in the NP laboratory revealed rare particles which, on account of their spherical shape and average diameter, can be considered similar to the NP produced. EDS analysis of these spherical particles (Figure 5, line scan mode) clearly shows O and Si signals, as expected. The Au signal is not detected probably because it is below the limit of detection (*LOD* 0.2–0.4 wt %) so we have no evidence of the Au nanostructures inside the SiO_2_ shell.

## 3. Discussion

The OECD approach serves to evaluate the results at the end of each tier, starting from specific decision criteria, balancing the costs and effectiveness of the exposure measurement strategy, in order to decide whether or not to proceed with the next tier. These criteria are based on comparison of the parameter values measured during the NOAA process and the corresponding background, and were further analyzed by Brouwer et al. [25]. The PNC measured during the NOAA process was considered statistically significant if it was higher than the background concentration plus three times the σ bkg [26,27].

The NF background may be influenced by previous nano-related processes run in the same laboratory and the concentrations may vary substantially from day to day. For these reasons, most studies so far have combined the NF and FF approaches and compared background with activity concentrations or simply subtracted the background from activity-related measurements [11]. If we apply these criteria to our study the mean bkg_FF_ plus three times the corresponding standard deviation is always below the mean of the bkg_NF_ obtained in the G, NW and NP laboratories. Therefore, we can assume these bkg_NF_ values as significant, above which the signal recorded by the CPC can probably be attributed to the NOAA production process: respectively 3990, 3340 and 2290 #/cm^3^ for the G, NW and NP laboratories.

On the basis of these considerations we can analyze the measurement features that exceed the significant values, in order to highlight possible NOAA dispersion during production processes. 

Phases 1 (sample preparation and chemical vapor deposition (CVD) reactor opening) and 3.1 (CVD reactor cleaning) of the process in the G laboratory show no peak intensity attributable to G production. Moreover, the process phase 1 shows decreases in PNC that might be related to a pure nitrogen flux originating from the CVD reactor during the vacuum chamber opening phase, which tends to lower the particulate concentration in the area of the laboratory where air is sampled. 

For process phases 2 (CVD growth) and 4 (cleaning the CVD reactor components in the furnace), it is interesting that the variations in the PNC are associated with events unrelated to the G production; as also reported in the workbook, these events can be traced back to the laboratory door being opened or closed, or related to staff movements and/or room cleaning.

The process phase 3.2, related to graphite spraying on some reactor components, shows a peak whose intensity is well above the significant value of PNC in the G laboratory; this peak was not associated with the G production process but was directly related to the graphite spraying. In addition, the off-line SEM investigations do not indicate any G particles on the filters collected by the Sioutas.

In the NW laboratory the CPC real-time measurements showed no changes in PNC associated with phase 3 (sample cleavage in the glove box). It was also evident that in the process phase 1 (chemical beam epitaxy (CBE) growth) there were features whose intensity was well above the significant value established for this laboratory; this high intensity might be associated with events not related to NW production, as mentioned for the G case study, because the NW growth occurs in a closed reactor under vacuum. Otherwise, during phase 2 (sample loading/unloading in the CBE load lock and sample mounting/unmounting in the glove box) there was a clear increase of the concentrations in the operator’s personal breathing zone (PBZ)—as defined by the European Committee for Standardization (CEN) [28]—although the PNC was always below the significant value established for the NW laboratory. The sample mounting/unmounting phase was also studied in the period between 3:58 p.m. and 4:07 p.m., introducing the sampling probes of instruments inside the glove box, and the measured PNC were high (Figure 3b, inset). This increase may be associated with the specific process task that involves heating the In on the molybdenum (Mo) sample holder during the mounting/unmounting of the sample on the hot plates [29,30]. However, in this case too, SEM did not indicate the presence of NW on the Sioutas filters. Although in our opinion this emission was probably related to sublimation of metallic In nanoparticles from the hot sample holder, a deeper investigation may be required to identify clearly the source and characteristics of the particulate emission during phase 2 of the process.

In the NP laboratory during a small part of the process phases 1 (synthesis in liquid) and 4 (drying) the PNC signal exceeded the significant value for this laboratory. Even sub-phase 4.1 (vacuum pump turned on/off), in which there was a fast change in PNC (Figure 3c, inset), passed the level of significance. Furthermore in the majority of phases 2 (aggregation in liquid) and 3 (shell building in liquid) the PNC were higher than the significant level.

**Figure 6:**
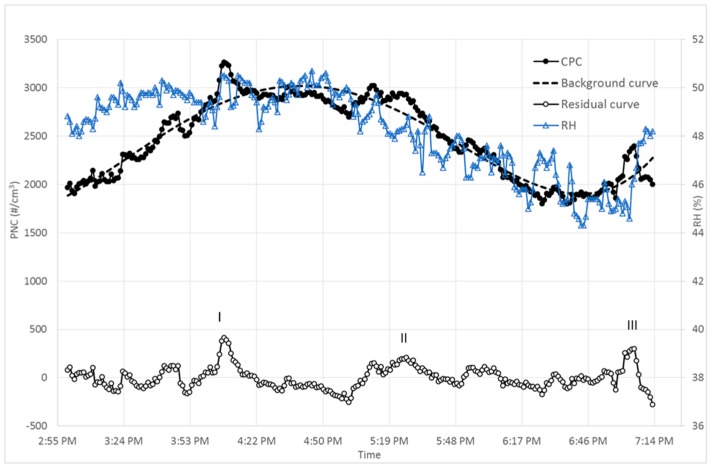
NP laboratory: PNC curve (solid black line + solid dots), background curve (dashed black line) and residual curve (black line + empty dots) as the difference between the PNC curve and the background curve, and the relative humidity curve (blue line + triangles).

In this case, the background contribution heavily influenced the PNC as measured by CPC in the NP laboratory. Compared to the previous cases, a different type of analysis can be proposed to take account of the background contribution: this would be to extrapolate the background contribution. In addition we compared CPC time series to microclimatic parameters measured by the BABUC-A probes, with particular reference to the relative humidity (RH) throughout the sampling period. After about 60 min from starting the measurement, the background contribution curve was strongly correlated with the RH curve, as clearly shown in Figure 6. This might be connected to nucleation phenomena, where high PNC values are associated with high RH—and vice versa [31,32].

The “residual” curve in Figure 6, obtained by subtraction of the background contribution curve from the PNC curve, shows essentially three structures: the first two need further clarification because they are not associated with any events recorded in the daily workbook, while the third corresponds to phase 4.1 (drying vacuum pump turned on) already shown in the inset of Figure 4c. This PNC increase is probably related to the pump emission.

In the case of NP, FE-SEM analyses indicated the presence of rare spherical particles similar in shape to the hollow SiO_2_ shells; however, the EDS probe did not detect the Au signal and there was no evidence of Au nanostructures in the FE-SEM image, probably because of the small amount of metal in each core shell. EDS microanalysis is a powerful and useful form of elemental analysis; however, it suffers some limitations: the limit of detection is generally above 1000–3000 ppm, but can vary for different elements, matrixes and analytical lines, and the spatial resolution is usually above 1 μm^3^ [33]. Therefore, the average gold fraction in the silica shell (<10% *w*/*w*) is too small to be detected and spatially resolved by the EDS micro-probe. However, since the EDS Au signal was also weakly detected in NP trial samples (see Section 2.1 and Figure A1 in Appendix), closer investigation will be required to make sure the sampled NPs are actually those produced or are generated by a different source of workplace air contamination, and, if possible, in which production phase this NP release may occur.

## 4. Materials and Process Descriptions 

These three case studies cover issues related to the nanoscale size and the aspect ratio, as crucial parameters for risk analysis. In particular, 2D G shows novel properties and has an increasing impact on product development and market prospects so methods for occupational safety and health (OSH) risk management are needed. Semiconductor 1D nanowires may also raise critical issues for OSH on account of their threadlike shape and similarities with hazard questions related to fibers. Silica 0D NP are widely used and already included in a range of nano-enabled consumer products, with an increasing impact on exposed populations.

**Figure 7:**
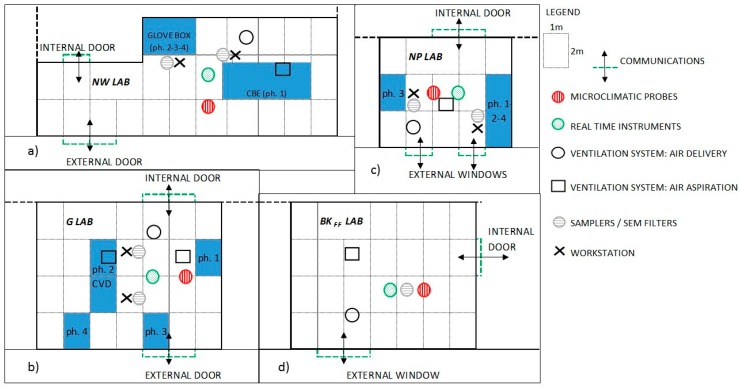
Schematic maps of the NW lab (**a**), G lab (**b**), NP lab (**c**) and Bkg_FF_ lab (**d**), including the places where each phase (ph.) of the three processes was done and the location of the FF background collection.

The different production processes conducted in the three R&D laboratories (Figure 7) at the same research facility are summarized below. Further information is reported in the technical data sheets of each material in Table A1.

### 4.1. 2D Graphene (G)

The process can be summarized in the following phases.

1. Sample preparation and loading. The reactor chamber is vented and the reactor lid is lifted manually; the sample (up to 10 × 10 mm) is placed on the graphite heater inside the reactor; the chamber is closed and pumped up to 5 × 10^−1^ mbar before starting a process.
2. CVD Growth. The growth process can be divided into two steps, both conducted in a commercial resistively heated cold-wall reactor (Aixtron HT-BM):
  2.1. Hydrogen etching. SiC substrates are treated with hydrogen etching at a temperature of around 1200 °C and a pressure of 450 mbar for a few minutes, in order to remove polishing scratches and obtain atomically flat terraces.
  2.2. Thermal decomposition. The hydrogen etched substrates are heated in an argon atmosphere at a temperature above 1300 °C and a pressure of 780 mbar for 10–15 min.
3. Reactor cleaning. The quartz and ceramic parts are periodically cleaned in an oven operated in air, in order to remove any carbon deposit. During the cleaning some reactor components are restored by graphite spraying.
4. Cleaning in the furnace. The parts are heated at 950 °C for at least one hour.

Each cycle produces a few tens of micrograms of G (with a total of a few milligrams per year). The G laboratory has an area of 40 m^2^ (about 120 m^3^ volume), with a mechanical ventilation system producing an air change of 3–6 volumes per hour. Phases 2 and 4 are run in a closed system. The process involves two or three workers equipped with personal protective devices (gloves, clothing and masks).

### 4.2. 1D Nanowires (NW)

NW production can be summarized in the following phases:

1. CBE growth. NW are synthesized through epitaxial growth techniques, e.g., the CBE, on a macroscopic crystalline substrate made of a semiconductor material, Si or InAs [30]. Usually, one end of the nanowire (the one not attached to the substrate) is composed of an Au metallic nanoparticle, as a semi-sphere with the same diameter as the NW, employed as catalyst during the growth process. The pressure achieved in the growth chamber is around 10^−10^ Torr.
2. Sample loading. This phase is divided into two following steps:
  2.1. Sample mounting and loading (before CBE growth). The substrate is first cleaved into small pieces of about 1 cm × 1 cm, and then fixed on a sample holder made of Mo through In-bonding inside a glove box. After that, the sample holder is placed in a cassette and transferred into the CBE system via a load-lock. The load-lock is pumped by a turbo-molecular pump and a base pressure of 10^−8^ Torr can be achieved in 1–2 h. The cassette is then transferred into the preparation chamber, and after that to the growth chamber, for NW synthesis.
  2.2. Sample unloading and unmounting (after the CBE growth). The plates with the grown samples are transferred from the growth chamber into the preparation chamber, mounted on the cassette that is transferred into the load-lock, and then again into the glove box, where the sample holder is placed on a hot plate at about 350 °C, to allow the In to melt, and the sample can be removed from the Mo plate. Frequently, a new sample mounting and loading (phase 2.1) is done immediately after the sample removal (phase 2.2).
3. Sample cleavage. The next phase is cleavage of the sample, for its morphological characterization by SEM.

The CBE chamber needs periodical maintenance, to clean the different parts of the system and inside the reactor. This is done by highly qualified staff, at least once a year.

Each cycle produces about 20 μg of NW with an estimated total laboratory production of 15 mg/year. The NW laboratory has an area of 20 m^2^ (about 60 m^3^ volume), with a mechanical ventilation system producing an air change of 3 volumes per hour; an automatic system for aspiration/cleaning starts in case of emergency. Phase 1 is in a closed system (ultra-vacuum chamber); phases 2–3 are carried out in a ventilated glove box. Two or three workers equipped with personal protective devices (gloves, clothing, FFP3 masks with filters for organic compounds) are involved in the process.

### 4.3. 0D Nanoparticles (NP)

Four different phases of the NP production process are:

1. Synthesis. The nano-architectures are synthesized by a wet chemical approach. A yellow solution of chloroauric acid underwent fast reduction by sodium borohydride in the presence of poly(sodium 4-styrene sulfonate) (PSS), with vigorous stirring, resulting in a deep orange colloidal solution of negatively charged gold NP, less than 3 nm in diameter.
2. Aggregation. The 3 nm gold NP are then assembled in spherical arrays by controlled aggregation achieved by ionic interaction with positive poly(l-lysine) (PL).
3. Shell building. The arrays are purified by cycles of centrifugation and silica-coated by a modified Stöber method [34]. The resulting products are “passion fruit-like” nano-architectures averaging 100 nm in diameter, with 20 nm wall thickness, and containing 1–10% *w*/*w* of metal.
4. Drying. The colloidal solutions were usually frozen in liquid nitrogen and freeze-dried overnight to obtain a red powder (about 1 mg for each synthesis with an estimated total laboratory production of 1 g/year).

The NP laboratory has an area of 30 m^2^ (about 90 m^3^ volume), with a mechanical ventilation system producing an air change of 3 volumes per hour. Phases 1 and 4 are carried out in a chemical ventilated hood. One worker equipped with personal protective devices (gloves, clothing and glasses) is involved in the process.

## 5. Methods

A measurement strategy based on a harmonized tiered approach [18] has been developed. The first tier, related to information gathering, was conducted in cooperation with the research laboratories involved in the NOAA production, by compiling a technical data sheet for each process (Table A1). On the basis of that information, a qualitative risk analysis was done, using the CB approach [21]. A small amount of the same materials produced in the three case studies (G, NW and NP) was provided by the laboratories, as a trial for the set-up of characterization methods. Since the release of NOAA cannot be excluded, the next tier of investigation was required.

The second tier included workplace walkthrough to obtain information about facilities, equipment, natural and mechanical ventilation and air change systems, production processes and phases, working times, collective and personal protective devices. Measurements were taken and air sampled. The instruments included easy-to-use and hand-held real-time devices, personal samplers and electronic microscopy for off-line analysis:

- Condensation particle counter (CPC mod. 3007, TSI Inc., Shoreview, MN, USA) to measure in real-time the number concentration (#/cm^3^) of particles from 10 nm to 1 µm, with 1 s time resolution and accuracy ±20%; (total flow 0.7 L/min; detection limits 1 to 100,000 #/cm^3^).
- Personal samplers (mod. Sioutas, SKC Inc., Eighty Four, PA, USA) equipped with a pump (mod. Leland Legacy, SKC Inc., 9 L/min flow) for concentrations (#/cm^3^) of particles from 250 nm to 2.5 µm (five stages).
- Field emission scanning electron microscope (FE-SEM) Ultra Plus (ZEISS) with EDS probe Inca 250-X-Max50 and INCA mapping software in line scan mode. The microscope is equipped with a modified Gemini column and has four different detectors: in-chamber Everhat–Thornely SE for surface topography, in-column In lens best for high efficiency, angle-selective backscattered detector ASB for material contrast and topographical information, in-column ESB for material contrast even at low KV.
- Microclimatic probes integrated in a control unit (BABUC-A, Lsi-Lastem Inc., Milano, Italy) to measure in real time physical parameters such as temperature, RH and air speed.

The third tier consisted of an extensive measurement campaign with real-time instruments and time-integrated samplers (for personal and area sampling). The samples were then fully analyzed off-line. The instruments included the same set-up as for Tier 2 with the following added devices:

- Fast mobility particle sizer (FMPS mod. 3550, TSI Inc.) to characterize real-time size distribution and simultaneously measure total particle numbers and mass concentration, in the size interval 5.6–560 nm, with 1 s time resolution;
- Nanoparticle surface area monitor (NSAM mod. 3091, TSI Inc.) to measure average and cumulative surface area (µm^2^/cm^3^) of particles from 10 nm to 1 µm, with 1 s time resolution, corresponding to the tracheobronchial (TB) or alveolar (A) pulmonary fractions, based on the model published by the International Commission on Radiological Protection [35];
- PAS 2000 (EcoChem Analytics, League City, TX USA) to measure polycyclic aromatic hydrocarbons (PAHs) surface-adsorbed on carbon aerosol with aerodynamic diameter from 10 nm to 1.5 µm, with a response time of 10 s in a measuring interval from 0 to 1000 ng/m^3^ and a lower detection limit of 3 ng/m^3^.
- Ozone analyzer (mod. 49, Thermo Environmental Instruments Inc., Franklin, MA, USA) to measure ozone levels in the air.
- Nano micro orefice uniform deposit impactor (nanoMOUDI-II 122R, MSP Corp., Shoreview, MN, USA), equipped with a rotary pump (BUSH LLC., Virginia Beach, VA, USA, 30 L/min flow), to collect particles in a dimensional range from 10 nm to 10 µm.
- Inductively coupled plasma mass spectrometry (ICP-MS 820, Bruker Corp., Billerica, MA, USA).
- Inductively coupled plasma optical emission spectroscopy (ICP-OES Agilent 5100, Agilent Technologies, Santa Clara, CA, USA).
- Atomic fluorescence spectrometer (AFS Titan 8200, Beijing Titan Instruments Co., Beijing, China).

An intensive measurement campaign was conducted, including all real-time and time- integrated instruments listed (whose main specifications are summarized in Table 2) and including tiers 2 and 3 for each case study. This option was allowed by the OECD methodology [18] that notes the possibility of approaching one, two or all tiers concurrently. As already mentioned, in line with the aim of the present study, we report here the main results of tiers 1 and 2 of the measurement strategy. The results of the investigation tier 3 will be the subject of further specific publications.

Instruments for area measurements and sampling were positioned inside the room within a range of 1.5 m around the workstation, taking into account possible influences from natural and forced ventilation systems. In some specific cases, the sampling tubes were placed in the worker’s PBZ or inside the glove-box (in the NW laboratory). Workers wore the personal samplers during their activities (Figure 7).

Measurements and sampling were done on two days for each case study: day 1–2 NW (Figure 7a), day 3–4 G, with an appendix on day 7 to measure the end of cleaning with the opening of the furnace (Figure 7b); day 7–8 NP (Figure 7c).

A room with the same volume, orientation, and structural and ventilation properties as the laboratories was selected for the indoor FF background characterization (Figure 7d): FF background measurements were made on day 5–6 when no work was ongoing. NF background was measured in each case study laboratory before each process. On day 9 real-time measurements of outdoor background were recorded.

Time-sheets of measurements in chronological order associated with each single phase of the processes and any further external event that arose during the activities were all entered in the daily register (study workbook).

Table 3 summarizes the time sheet of the full measurement campaign.

## 6. Conclusions

We studied three exposure scenarios of production of NOAA with different dimensionalities—2D G, NW with a 1D structure and NP with a 0D shape, applying tiers 1 and 2 of the OECD harmonized methodology [18], to assess whether these levels of analysis gave effective results. We investigated the real-time PNC measured by CPC in addition to the microclimatic parameters and the off-line FE-SEM coupled with EDS analysis, in order to balance the costs related to the instruments involved with the reliability of the results, as recommended by the OECD guideline. FE-SEM with high resolution successfully characterized the NP and the results were comparable to the capabilities of transmission electron microscopy (TEM).

We also looked closely into the background characterization in order to identify the significant PNC values for each laboratory. The FF background was measured in a room with structural characteristics similar to the study laboratories, during two non-working days. We adopted this approach as an alternative to simultaneous measurement of FF background and NOAA in order to overcome limitations related to the availability of instruments; bkg_FF_ values were compared to bkg_NF_ measured before starting the NOAA production in the three laboratories. Since the bgk_FF_ was always lower than the bkg_NF_ in all three laboratories, we took the bkg_NF_ values plus three times the corresponding standard deviations as significant, above which the CPC measurement can be attributed to the NOAA production process.

The σ bkg_NF_ values were different in the three laboratories and this variability was probably influenced by the frequent access of research staff for work-related activities in the laboratory and near the CPC sampling point during the background data collection interval; this particular aspect may need further specific analysis.

In the NP laboratory we examined a different approach to assess the background contribution because the PNC measured by CPC was closely correlated to the RH. In this respect, it will be interesting to clarify the relations between background PNC and microclimatic parameters, as a recommendation for further research.

In general, we concluded that to completely characterize the exposure scenarios of NOAA production in the three case studies, additional resources in terms of instrumentation and a more detailed analysis (as envisaged by the OECD in tier 3) will be required; this might imply considering the size distribution, the surface areas of the particulate matter, the concentrations of the PAHs and other pollutants and, finally, chemical and morphological analysis using TEM on ad hoc collected samples.

Specific findings for each case study are reported as follows:

- G laboratory. Real-time measurements showed high PNC associated with phase 3.2 of graphite spraying during the CVD reactor cleaning, but SEM did not detect any produced G on the sampled filters. As a precautionary measure, this specific phase should be conducted under an aspiration hood or in a glove box.
- NW laboratory. Although the CPC measurements showed a rise in PNC during phase 2 (sample mounting and loading/unloading and unmounting), SEM did not detect any NW on the Sioutas filters. Although the PNC was probably related to sublimation of In from the hot Mo plate, an ad hoc investigation is called for to properly analyze the chemical and physical parameters of the emission source in this specific phase. In any case, the measurements made inside the glove box demonstrated the effectiveness of this containment measure. The study of the CBE reactor cleaning phase (not included in the present measurement campaign), might be useful to analyze the whole process so as to finally exclude workers’ exposure to NW.
- NP laboratory. In a large part of the process, PNCs were greater than the significant value identified for this laboratory. In this case the background contribution heavily influenced the PNC, as measured by CPC, so we proposed a different type of analysis to take account of the background. By comparison of CPC time series with microclimatic parameters measured with BABUC-A probes, with particular reference to the RH throughout the sampling period, we found a structure corresponding to phase 4.1 (drying vacuum pump turned on) that exceeded the background curve; the PNC increase in this case is probably related to the pump switching and not strictly connected to NP production. However, SEM of sampled filters showed there were rare spherical SiO_2_ particles (with an approximate diameter of 100 nm) similar to the hollow silica shells produced but with no evidence of the Au nanostructures inside. This implies that further investigation is needed to confirm whether such NPs are in fact released by the process and in which specific phase this release might occur.

In conclusion, also according to the findings of the complementary study on workers’ personal exposure conducted in the same research facilities [22] and to the broader objectives of the research project of which the present study is part, the results provide important information for improving risk management in laboratory workplaces.

The measurement strategy may be used to study workers’ exposure to NOAA also in industrial production processes, with promising applications in the near future. Its integration in a prevention-through-design model, taking into account the cost and effectiveness of workplace monitoring techniques, will contribute to the responsible development and use of nano-products.

## Figures and Tables

**Table 1:** Average and standard deviation of measured background concentrations (#/cm^3^).

Background Type	Collection Interval	Mean PNC (#/cm^3^)	σ bkg (#/cm^3^)
bkg_FF_	Day 5 (11:13 a.m.–6:15 p.m.)	948	127
bkg_FF_	Day 6 (9:30 a.m.–5:09 p.m.)	1023	136
bkg_FFavg_	Day 5–Day 6	986	167
bkg_outdoor_	Day 9 (10:51 a.m.–4:29 p.m.)	6554	1519
bkg_NF-G_	Day 3 (10:10 a.m.–10:25 a.m.)	2966	258
bkg_NF-NW_	Day 1 (10:26 a.m.–10:41 a.m.)	2835	157
bkg_NF-NP_	Day 7 (2:57 p.m.–3:12 p.m.)	2005	95

**Table 2:** Main specifications of real-time and time-integrated instruments employed in the intensive measurement campaign. UV: ultraviolet; PAHs: polycyclic aromatic hydrocarbons; TB: tracheobronchial; A: alveolar.

Instrument	Class	Principle of Operation	Outputs	Size Range (nm)	Time Resolution (s)	Total Flow L/min	Detection Limits
CPC TSI Inc. Mod. 3007	Real-time device	Optical detection	Particle number concentration (#/cm^3^)	10–1000	1	0.7	1 to 100,000 #/cm^3^
FMPS TSI Inc. Mod. 3091	Real-time device	Electrical mobility	Particle number concentration (#/cm^3^) Size distribution	5.6–560	1	10	Small particles: 100–1 × 10^7^ #/cm^3^ Large particles: 1–1 × 10^5^ #/cm^3^
NSAM TSI Inc. Mod. 3550	Real-time device	Diffusion charging	Avg. (µm^2^/cm^3^) and Tot. (µm^2^) surface area of TB or A fractions	10–1000	1	2.5	TB: 0 to 2500 μm^2^/cm^3^ A: 0 to 10,000 μm^2^/cm^3^
O_3_ Analyzer TEI Inc. Mod. 49 C	Real-time device	UV photometric measurement	Ozone conc. (ppb)	-	20	1–3	>1 ppb
PAS2000 EcoChem Inc.	Real-time device	Photoelectric Ionization	PAHs (ng/m^3^)	10–1000	10	2	>3 ng/m^3^
nanoMOUDI MSP Mod. 122 R	Time-integrated device: Area Sampler	Aerodynamic diameter	Particle gravimetric mass Size distribution Samples for off-line analysis	10–18,000	-	30	-
Sioutas	Time-integrated device: Personal sampler	Aerodynamic diameter	Particle gravimetric mass Size distribution Samples for off-line analysis	250–2500	-	9	-

**Table 3:** Time sheet of measurements during the three case studies.

Day/Hours	Process/Phase	Day/Hours	Process/Phase
**Day 1**	**Nanowires**	**Day 2**	**Nanowires**
10:26 a.m.–10:41 a.m.	0. Background NF	9:40 a.m.–9:55 a.m.	0. Background NF
10:41 a.m.–12:05 a.m.	1. CBE Growth	9:56 a.m.–11:45 a.m.	1. CBE Growth
12:07 a.m.–12:33 a.m.	2. Sample Loading	12:00 a.m.–12:09 a.m.	2. Sample Loading
12:25 a.m.–3:15 p.m.	1. CBE Growth	12:18 a.m.–12:19 a.m.	3. Sample Cleavage
2:38 p.m.–2:49 p.m.	2. Sample Loading	1:15 a.m.–3:25 p.m.	1. CBE Growth
3:15 p.m.–4:35 p.m.	1. CBE Growth	1:35 p.m.–1:41 p.m.	2. Sample Loading
4:45 p.m.–5:00 p.m.	2. Sample Loading	1:42 p.m.–1:45 p.m.	3. Cleavage
5:00 p.m.–6:10 p.m.	0. Background NF	3:58 p.m.–4:07 p.m.	2. Sample Loading ^1^
		4:08 p.m.–4:09 p.m.	3. Sample Cleavage ^1^
		4:26 p.m.–5:09 p.m.	0. Background NF
**Day 3**	**Graphene**	**Day 4**	**Graphene**
10:10 a.m.–10:25 a.m.	0. Background NF	10:05 a.m.–10:20 a.m.	0. Background NF
10:25 a.m.–10:34 a.m.	1. Sample Preparation and Recator 1 Opening	10:20 a.m.–10:21 a.m.	1. Sample Preparation and Recator 1 Opening
10:34 a.m.–11:00 a.m.	2. CVD Growth	10:21 a.m.–12:05 a.m.	2. CVD Growth
11:00 a.m.–11:03 a.m.	1. Sample Preparation and Recator 1 Opening	10:45 a.m.–10:55 a.m.	1. Sample Preparation and Recator 2 Opening
11:03 a.m.–11:50 a.m.	2. CVD Growth	12:05 a.m.–12:12 a.m.	1. Sample Preparation and Recator 1 Opening
11:50 a.m.–11:53 a.m.	1. Sample Preparation and Recator 1 Opening	12:12 a.m.–12:33 a.m.	3.1 Cleaning
11:53 a.m.–12:49 a.m.	2. CVD Growth	12:27 a.m.–12:30 a.m.	3.2 Cleaning: Graphite Spraying
12:49 a.m.–12:53 a.m.	1. Sample Preparation and Recator 1 Opening	12:33 a.m.–2:00 p.m.	0. Background NF
12:53 a.m.–2:58 p.m.	2. CVD Growth	2:00 p.m.–2:30 p.m.	0. Background NF ^2^
2:58 p.m.–3:00 p.m.	1. Sample Preparation and Recator 1 Opening	2:30 p.m.–end of cycle (Day 5)	4. Cleaning in Furnace ^2^
3:00 p.m.–5:30 p.m.	2. CVD Growth		
5:30 p.m.–6:05 p.m.	0. Background NF		
**Day 5**	**Background**	**Day 6**	**Background**
11:48 a.m.–6:15 p.m.	0. Background FF	9:30 a.m.–5:09 p.m.	0. Background FF
**Day 7**	**Graphene**		
10:00 a.m.–10:05 a.m.	0. Background NF ^2^		
10:05 a.m.–10:10 a.m.	4. Cleaning: Opening The Furnace ^2^		
**Day 7**	**Nanoparticles**	**Day 8**	**Nanoparticles**
2:57 p.m.–3:12 p.m.	0. Background NF	9:33 a.m.–9:48 a.m.	0. Background NF
3:12 p.m.–3:33 p.m.	1. Synthesis	9:48 a.m.–10:10 a.m.	1. Synthesis
3:33 p.m.–3:45 p.m.	2. Aggregation	9:53 a.m.–9:55 a.m.	4.1 Drying: Vacuum Pump Turned off (NP produced during day 7)
3:45 p.m.–7:00 p.m.	3. Shell Building	10:10 a.m.–10:25 a.m.	2. Aggregation
7:00 p.m.–7:05 p.m.	4.1 Drying: Vacuum Pump Turned on	10:25 a.m.–1:26 p.m.	3. Shell Building
7:05 p.m.–7:19 p.m.	4.2 Drying	1:26 p.m.–1:29 p.m.	4.1 Drying: Vacuum Pump Turned oN
		1:29 p.m.–3:24 p.m.	4.2 Drying
		3:24 p.m.–3:28 p.m.	4.1 Drying: Vacuum Pump Turned off
		4:25 p.m.–5:29 p.m.	0. Background NF
**Day 9**	**Background**		
9:40 a.m.–5:28 p.m.	0. Background Outdoor		

^1^ CPC and FMPS placed inside the glove box; ^2^ CPC and FMPS placed close to the furnace.

**Figure A2:**
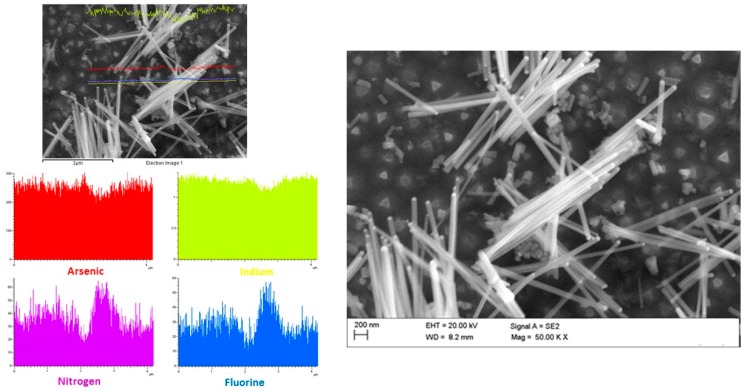
Nanowires (NW).

## Appendix A

**Table A1:** Technical Data Sheets of Three Nanomaterials: Nanoparticles (NP), Nanowires (NW), and Graphene (G). n.a.: not available.

Technical Data Sheets	NP	NW	G
1. NOAA information			
1.1 Technical name and/or commercial name	Hollow silica nanoparticles with embedded gold nanoparticles (AuSiO_2_)	Semiconductor nanowires (NW)	Graphene
1.2 CAS Number	CAS number Au: 7440-57-5 CAS number SiO_2_: 7631-86-9 CAS number Poly-l-lysine hydrobromide 15–30 kDa: 25988-63-0 CAS number Poly(sodium 4-styrenesulfonate) 70 kDa: 25704-18-1	n.a. (CAS number InAs)	n.a. (CAS number Carbon)
1.3 Molecular structure/Crystal structure	100 nm silica (SiO_2_) nanocapsules containing: (i) 30–500 gold nanoparticles with diameters 2–4 nm; (ii) poly(l-lysine) 15–30 kDa; (iii) poly(sodium 4-styrenesulfonate) 70 kDa	Crystal structure: mainly hexagonal (Wurzite) with cubic insertions (Zincoblende). NW have their long axis oriented along the hexagonal c-axis which is parallel to the cubic <111> direction	Single atomic layer of carbon atoms disposed in a honeycomb lattice
1.4 Chemical composition (including surface compounds)	The powders are 5% gold and 95% silica (SiO_2_) and organic polymers.	InAs (50/50) with a top Au_0.5_In_0.5_ nanoparticle (eutectic).	Carbon
1.5 Physical form and shape	Spherical nanoparticles	NW are rod-shaped crystals, with hexagonal cross-section and a AuIn nanoparticle attached at one end.	Bi-dimensional crystal taking the form of flakes
1.5.1 Common form	The common forms are: (i) powder (after freeze-drying) (ii) in water solutions	NW grow normal to the InAs (111) substrate surface, forming a “forest” connected to the substrate at one end.	Single crystals with dimensions from microns to millimeters
1.6 Surface chemistry	Negative charge (−21 V in PBS)	InAs	Carbon
1.7 Production method	Wet chemical synthesis	Chemical Beam Epitaxy (CBE) synthesis	Chemical Vapor Deposition (CVD) synthesis
2. NOAA Characterization			
2.1 Aggregation/Agglomeration	Single nanoparticles in solutions. Possible aggregation after freeze-drying	NW can agglomerate if detached from the substrate	If detached from the substrate they can aggregate
2.2 Solubility	Tested, up to 100 mg/mL in aqueous solutions	Insoluble in bases, organic solvents and biological media. Soluble in acids.	Non soluble
2.2.1 Dispersibility	Up to 100 mg/mL in ethanol	NW can be dispersed in liquid (i.e., 2-propanol) via sonication or by mechanical transfer from the substrate.	Can be dispersed in organic solvents
2.3 Crystal phase	n.a.	NW are single crystals with a lattice parameter of 6.0583 Angstrom	Hexagonal bi-dimensional lattice
2.4 Dustiness (or bulk material density)	n.a.	Bulk InAs density: 5.67 g/cm^3^	n.a.
2.5 Representative image (SEM)	See attached Figure A1	See attached Figure A2	In Convertino et al. [36]
2.6 Size	100 ± 20 from TEM images (analysis of at least 300 nanoparticles)	NW can be synthetized with average diameters in the 30–100 nm range, and lengths of 1–2 μm. Dimensions are typically obtained from SEM images.	Graphene flakes have lateral dimensions that vary from fraction of microns to millimeters
2.7 Surface area	About 30,000 nm^2^/nanostructure	300,000 nm^2^ (1 NW)	n.a.
2.8 Catalytic or photocatalytic activity	No	No	n.a.
2.9 Density	n.a.	Average substrate area density of NW ranges from 1 to 250 NW per μm*^2^*. Area density is determined from SEM images. If left attached to the substrate the area density is indicated as above. Concerning the “mass per unit volume” density of the NW layer on the substrate surface (considering “typical” 60 nm in diameter and 1 μm length NW with an area density of 50 NW/μm^2^): it is a 1 μm thick layer covering the whole substrate surface with normal-to-the-substrate NW. This layer has an average density of 0.8 g/cm^3^	n.a.
2.10 Porosity	n.a.	No	n.a.
2.11 Surface reactivity	Covalent bonding with silanols (ex. APTES). Possible adsorptions of positive molecules	Little reactivity	n.a.
2.12 Other information	n.a.	n.a.	n.a.
3. Processes			
3.1 Average quantity produced/used per year	1 g	20 mg	2–3 mg
3.2 Average quantity produced/used in each process	1 mg	40 μg	1 µg
3.3 Process phases description	Synthesis. The nano-architectures are synthesized by a wet chemical approach. A yellow solution of chloroauric acid underwent fast reduction by sodium borohydride in the presence of poly(sodium 4-styrene sulfonate) (PSS), with vigorous stirring, resulting in a deep orange colloidal solution of negatively charged gold NP, less than 3 nm in diameter. Aggregation. The 3 nm gold NP are then assembled in spherical arrays by controlled aggregation achieved by ionic interaction with positive poly(l-lysine) (PL). Shell building. The arrays are purified by cycles of centrifugation and silica-coated by a modified Stöber method [34]. The resulting products are “passion fruit-like” nano-architectures averaging 100 nm in diameter, with 20 nm wall thickness, and containing 1–10% *w*/*w* of metal. Drying. The colloidal solutions were usually frozen in liquid nitrogen and freeze-dried overnight to obtain a red powder (about 1 mg for each synthesis with an estimated total laboratory production of 1 g/year).	CBE growth. NW are synthesized through epitaxial growth techniques, e.g., the CBE, on a macroscopic crystalline substrate made of a semiconductor material, Si or InAs [30]. Usually, one end of the nanowire (the one not attached to the substrate) is composed of an Au metallic nanoparticle, as a semi-sphere with the same diameter as the NW, employed as catalyst during the growth process. The pressure achieved in the growth chamber is around 10^−10^ Torr. Sample loading. This phase is divided into two following steps: 2.1 Sample mounting and loading (before CBE growth). The substrate is first cleaved into small pieces of about 1 cm × 1 cm, and then fixed on a sample holder made of Mo through In-bonding inside a glove box. After that, the sample holder is placed in a cassette and transferred into the CBE system via a load-lock. The load-lock is pumped by a turbo-molecular pump and a base pressure of 10^−8^ Torr can be achieved in 1–2 h. The cassette is then transferred into the preparation chamber, and after that to the growth chamber, for NW synthesis. 2.2 Sample unloading and unmounting (after the CBE growth). The plates with the grown samples are transferred from the growth chamber into the preparation chamber, mounted in the cassette that is transferred into the load-lock, and then again into the glove box, where the sample holder is placed on a hot plate at about 350 °C, to allow the In to melt, and the sample can be removed from the Mo plate. Frequently, a new sample mounting and loading (phase 2.1) is done immediately after the sample removal (phase 2.2). Sample cleavage. The next phase is cleavage of the sample, for its morphological characterization by SEM. The CBE chamber needs periodical maintenance, to clean the different parts of the system and inside the reactor. This is done by highly qualified staff, at least once a year.	Sample preparation and loading. The reactor chamber is vented and the reactor lid is lifted manually; the sample (up to 10 × 10 mm) is placed on the graphite heater inside the reactor; the chamber is closed and pumped up to 5 × 10^–1^ mbar before starting a process. CVD Growth. The growth process can be divided into two steps, both conducted in a commercial resistively heated cold-wall reactor (Aixtron HT-BM): 2.3 Hydrogen etching. SiC substrates are treated with hydrogen etching at a temperature of around 1200 °C and a pressure of 450 mbar for a few minutes, in order to remove polishing scratches and obtain atomically flat terraces. 2.4 Thermal decomposition. The hydrogen etched substrates are heated in Argon atmosphere at a temperature above 1300 °C and a pressure of 780 mbar for 10–15 min. Reactor cleaning. The quartz and ceramic parts are periodically cleaned in an oven operated in air, in order to remove any carbon deposit. During the cleaning some reactor components are restored by graphite spraying. Cleaning in the furnace. The parts are heated at 950 °C for at least one hour.
3.4 Workplace description	The NP laboratory has an area of 30 m^2^ (about 90 m^3^ of volume)	The NW laboratory has an area of 20 m^2^ (about 60 m^3^ of volume)	G laboratory has an area of 40 m^2^ (about 120 m^3^ of volume)
3.4.1 Other processes in the same workplace	The synthesis are performed in the lab where are also performed chemical synthesis and purifications of organic molecules.	Substrates with NW can be cleaved inside the negative pressure glove box to do SEM observation or other characterization. During SEM observation (taking place in an ISO6 cleanroom) o additional processes take place.	n.a.
3.4.2 Ventilation system	Mechanical ventilation system producing an air change of 3 volumes per hour	Mechanical ventilation system producing an air change of 3 volumes per hour; in case of emergency an automatic system for aspiration/cleaning starts.	Mechanical ventilation system producing an air change of 3–6 volumes per hour
3.5 Number of workers	1	2–3	1–2
3.7 Avg duration of production process	5 h	2 h	2 days
3.8 Avg working days per year	250	230	n.a.
3.9 Number of production processes per day	0–6	3–4	n.a.
3.10 Risk assessment method and results	Control Banding: Medium	Control Banding: High	Control Banding: High
3.11 Exposure measurements/monitoring	n.a.	n.a.	n.a.
3.12 Safety operating procedures	yes	yes	yes
3.13 Protective equipment	Phases 1 and 4 are performed within chemical ventilated hood. Worker equipped with personal protective devices (gloves, clothing and glasses).	Phase 1 is in closed system (ultra-high-vacuum reactor); phases 2–3 are performed in a ventilated glove box. Workers are equipped with personal protective devices (gloves, clothing, half- and full-face masks with A2B2E2K2P3 filters).	Phases 2 and 4 are fulfilled in a closed system. Workers are equipped with personal protective devices (gloves, clothing and masks).
4. References			
4.1 Main references	Voliani and Piazza [37], Cassano et al. [38]	Tomioka et al. [39], Gomes et al. [30], Rocci et al. [40].	Novoselov et al. [41], Convertino et al. [36], Miseikis et al. [42]
5. Other			
5.1 Any other available information	n.a.	n.a.	n.a.
